# Functional characteristics and habitat suitability of threatened birds in northeastern China

**DOI:** 10.1002/ece3.11550

**Published:** 2024-06-25

**Authors:** Jianwei Li, Haibo Jiang, Mingjun Xie, Chuantao Song, Chunguang He, Hongfeng Bian, Lianxi Sheng

**Affiliations:** ^1^ State Environmental Protection Key Laboratory of Wetland Ecology and Vegetation Restoration, School of Environment Northeast Normal University Changchun China

**Keywords:** Biomod2, bird distribution, climate impact, ecological characteristics, threatened species

## Abstract

Northeast China, rich in natural resources and diverse biodiversity, boasts a unique habitat for threatened bird species due to its remote location and perennial cold climate. An analysis assessed the adaptability of these species using data on their geographic distribution and functional traits collected through database queries. The results revealed that threatened bird species share similar functional traits and a stronger phylogenetic signal (Blomberg mean *K* = 0.39) compared to common species. The Biomod2 model analyzed potentially suitable ranges and environmental drivers under current and future climate scenarios, showing a pattern of larger suitable areas in southern regions and smaller suitable areas in the north. The most critically threatened species faced greater geographical constraints (0.989), with mean annual temperature being a key influence. Altitude and water system distribution were also key factors impacting the distribution of other threatened bird species. Simulated projections under different climate scenarios (RCP 45 and 85) indicated varying degrees of expansion in the suitable range for these species. This research sheds light on the functional traits and distribution of threatened bird species in Northeast China, providing a scientific foundation for future conservation and management efforts.

## Graphical abstract

This study assesses the adaptability of threatened bird species in Northeast China by analyzing their geographic distribution and functional traits. Utilizing the Biomod2 model, it predicts changes in suitable habitats under various climate scenarios, identifying key environmental drivers like temperature, altitude, and water systems. The research offers critical insights for conservation efforts, highlighting a stronger phylogenetic signal among these species and projecting habitat shifts due to climate change.

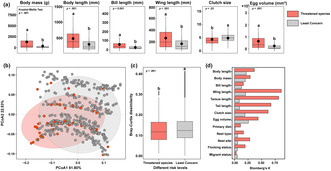

## 1 INTRODUCTION

Climate change is considered one of the primary threats to biodiversity and ecosystems (Ebrahimi et al., 2021). It is estimated that the current rate of species extinction is higher than at any time in the past 65 million years (Pimm et al., 2014). Understanding the population distribution and dynamic changes of threatened species is crucial for providing effective protection, especially in remote and underdeveloped regions of the world (Herrera‐Sánchez et al., 2020). These areas often lack sufficient surveys and monitoring, leading to limited knowledge about species facing extreme threats. This lack of information makes it challenging to formulate conservation strategies and management plans tailored to these species. Assessing the speed and extent of changes in the distribution range of threatened species is of utmost importance for the formulation of biodiversity conservation policies, the planning of protected areas, and ecosystem management.

Functional traits are reflective of the adaptive strategies that species have developed through long‐term interactions with their environment (Suding et al., 2008). These functional traits can encompass physiological features (e.g., metabolic rate, water use efficiency), morphological characteristics (e.g., body size, beak shape), or behavioral traits (e.g., feeding habits, migration patterns), all of which play a crucial role in the survival and reproduction of birds in specific environments (Dehling et al., 2014). In extreme environments, species face heightened physiological pressures for survival and reproduction, which may drive closely related species to develop consistent adaptive strategies, resulting in similar physiological or morphological adaptations (Cheng et al., 2021). For instance, birds inhabiting saline–alkali environments have evolved a set of physiological, behavioral, and morphological mechanisms to maintain constant intracellular and extracellular ion balance (Gutiérrez, 2014). Species living in arid regions may evolve higher water use efficiency and drought‐resistant physiological mechanisms to cope with scarce water conditions (Williams & Tieleman, 2002). Similarly, high‐altitude species may exhibit greater lung capacity and oxygen uptake ability to adapt to lower oxygen levels and cold climates (Laguë, 2017). These shared genetic responses may arise due to similar adaptive demands faced by species under common environmental selection pressures, indicating that birds may independently develop similar functional traits when confronted with similar environmental challenges. Such similar adaptive strategies are likely preserved and transmitted through evolutionary mechanisms like natural selection (Díaz et al., 2013). When bird species encounter similar threats or face comparable environmental conditions, they may experience parallel selection pressures, which could lead to the development of similar morphological adaptations (Loiseau et al., 2020; Sayol et al., 2021). Multiple species competing for the same resources in the same habitat may evolve similar morphological characteristics to reduce competition pressure and optimize resource utilization (Crouch & Tobias, 2022). Moreover, when bird species confront severe threats such as habitat loss, predation pressure, or human disturbances, they encounter shared survival challenges. Under such circumstances, natural selection may drive these species towards similar adaptive strategies, enhancing their chances of survival and reproductive success, possibly resulting in these threatened bird species displaying more similar traits (Waters et al., 2020).

The Northeast region of China is one of the country's most biodiverse areas, boasting diverse ecological environments and complex climate conditions. However, in recent years, the continuous growth of the population and intensifying human activities have exacerbated ecosystem degradation, leading to gradual habitat destruction (Xue et al., 2021). Overexploitation of biological resources has resulted in the gradual decline and even extinction of some environmentally sensitive species. Climate change has impacted the region's biodiversity, with some species migrating from their original habitats to new environments. During this migration process, certain sensitive species may face local extinction, and populations may become highly fragmented (Ghehsareh Ardestani & Heidari Ghahfarrokhi, 2021). Prolonged habitat fragmentation may deplete genetic variation and lead to elevated levels of inbreeding, posing a threat to the survival of the remaining populations (Klütsch et al., 2021). Functional diversity and uniqueness describe the characteristics of multi‐species that influence their performance and contribute to ecosystem functionality (Cox et al., 2023; Violle et al., 2017). In terms of species functional trait composition, “trait scarcity” quantifies the infrequency of traits at the local scale. It is specific to the survival strategies of species within their habitats, to avoid competition with other species, release intraspecific competition, or exploit alternative resources to expand their ecological niche width (“strategy”) (Violle et al., 2017). Changes in global conditions, such as bird reduction or local extinction, can lead to the loss of specific traits or functionalities associated with species in certain areas. “Threats” can affect multiple species with similar traits, resulting in the potential loss of limited functional diversity, influencing ecosystem functionality (Cadotte et al., 2011). Previous studies have found that threatened species appear to have higher trait similarity, supporting more irreplaceable unique traits or functionalities (Sayol et al., 2021). Threatened species are often geographically constrained, with relatively small distribution ranges. This geographic constraint may be due to various factors, including habitat destruction, fragmentation, habitat loss, and human disturbances (Loiseau et al., 2020). Geographic constraints can also lead to increased competition and predation pressure on threatened species (Jaatinen et al., 2022). Due to their limited distribution range, threatened bird species may encounter more competitors and predators in their habitats, thereby increasing competition pressure on their survival and reproduction.

Understanding species distribution and their habitat requirements is a crucial topic in ecology and conservation. Therefore, habitat suitability models or species distribution models (SDMs) have been used to describe the habitat or occurrence probability of bird species. Many SDMs address the issue of predicting species distribution under climate change scenarios, providing vital information for the conservation management of identified critical habitats (Cerasoli et al., 2022; Latimer et al., 2006). Habitat suitability models have various applications in conservation management. For example, they can aid in delineating natural reserves or protected areas to provide suitable living spaces for threatened species. By understanding the specific responses of birds and their sensitivity to climate change, critical habitats can be identified, and corresponding conservation measures can be implemented (Jaatinen et al., 2022). These models can also be used to assess the potential impact of climate change on biodiversity, facilitating the development of adaptive management strategies and conservation planning (Yang et al., 2018). Climate factors such as temperature and precipitation play a significant role in shaping the habitat range of different bird species. Climate changes are geographically uneven, leading to heterogeneity in species distribution changes, with some species showing more pronounced responses to local climate variations. Species traits vary due to multiple environmental factors (Zhang et al., 2017). Phenotypic and behavioral plasticity in response to climate variability reflects sensitivity or tolerance to environmental changes, and the heterogeneity in species distribution changes is closely related to local climate variations (Hoffmann & Bridle, 2022). Simulating species distribution under future climate change conditions can guide us in gaining deeper insights into the patterns of species distribution changes. Birds are one of the most extensively studied vertebrate groups, and we have gained a comprehensive understanding of their traits, distribution, and abundance. Furthermore, birds serve as excellent subjects for investigating species distribution trends because their ranges are closely associated with temperature and water distribution (Lei & Liu, 2021; Root, 1988).

In this study, we utilized the traits of different threatened bird species and the Biomod2 distribution model to explore the characteristics and distribution patterns of bird traits in a localized region, specifically the Northeast region of China. This region is located on the East Asia‐Australasia Flyway, where nearly 10 million migratory birds migrate and breed each year (Yong et al., 2021). Changes in regional bird habitat conditions have important implications for the conservation of birds in East Asia as a whole. The objectives of this research were as follows: (a) To examine the pronounced phylogenetic similarity among threatened bird species in terms of their traits. (b) Identify the unique functional diversity and geographic constraints of threatened birds in the Northeast and predict future changes in their distribution. By investigating the traits and distribution patterns of threatened bird species in the Northeast region, this study aims to contribute valuable insights into their ecological characteristics and provide essential information for conservation and management efforts in this ecologically significant area.

## 2 MATERIALS AND METHODS

### 2.1 Overview of the study area

**FIGURE 1:**
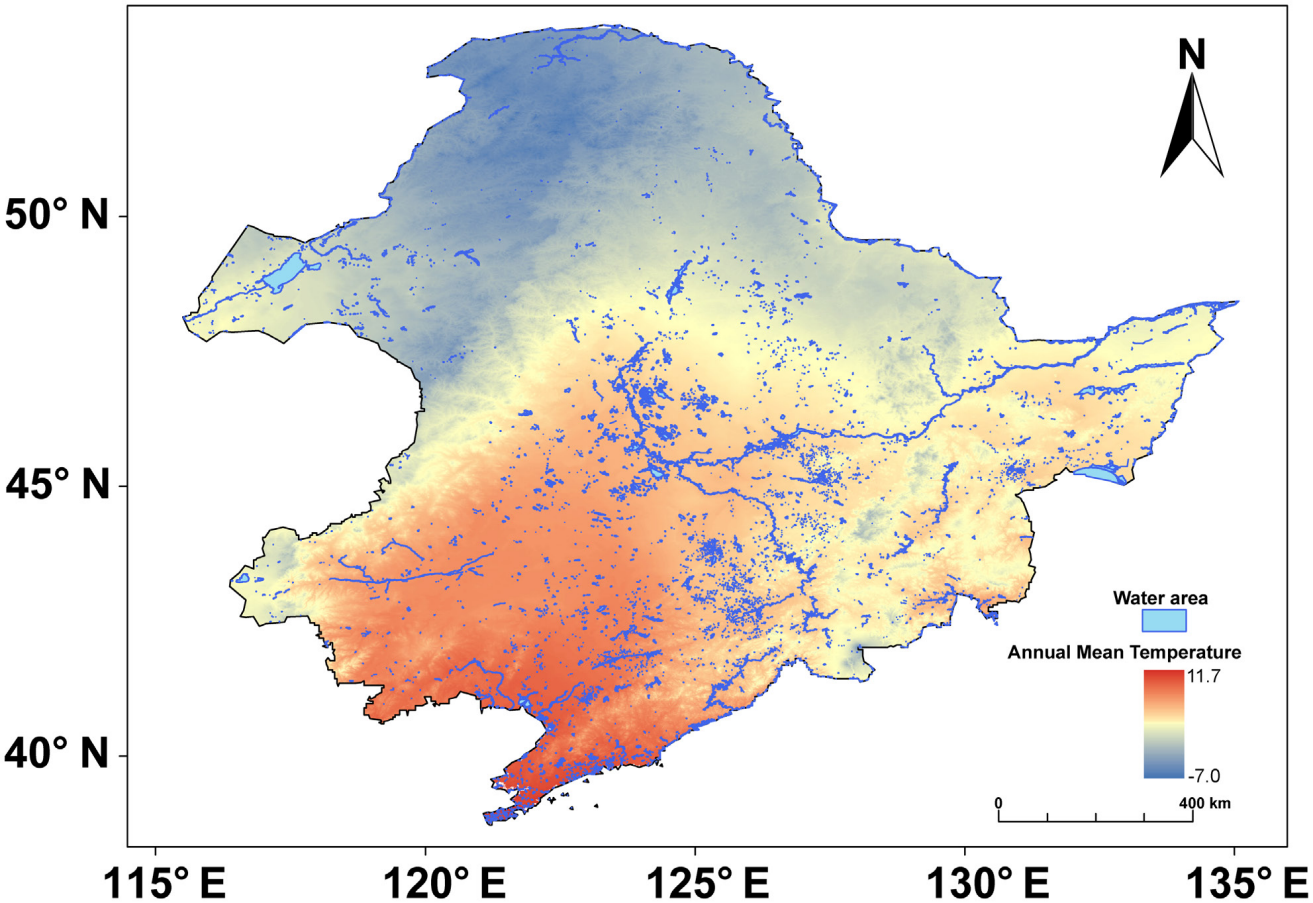
Annual temperature variation and water body coverage in the Northeast China region.

The northeastern region experiences four distinct seasons with a wide range of average annual temperatures, providing diverse habitats such as water bodies, wetlands, and forests, which provide abundant food and habitat resources for various bird species (Figure 1; −7 to 11.7°C). The study area covers the provinces of Heilongjiang, Jilin, Liaoning and northeastern Inner Mongolia in China. This region is mainly characterized by a temperate monsoon climate, with annual precipitation ranging from 400 to 1000 mm and an average annual temperature of −7 to 6°C (Yang et al., 2018). The area has lush vegetation with a tree cover of about 42.9%. The dominant vegetation types include *Pinus koraiensis*, *Larix gmelinii*, and *Betula platyphylla* (Yang et al., 2018). The topography of the region varies, with higher elevations surrounding lower areas. These diverse ecosystems support the survival of many wildlife species. The Northeast region is also home to the Songnen Plain and the Sanjiang Plain, both of which have well‐developed wetlands that are major habitats for threatened birds.

### 2.2 Acquisition of data

We collected data on the distribution of 463 bird species recorded in the Northeast region from the Global Biodiversity Information Facility (GBIF, http://www.gbif.org/), the eBird database (eBird, https://ebird.org/) and the China Bird Report (http://www.birdreport.cn/). We did a lot of comparing and screening, merging between different databases, and asking relevant experts to ensure the accuracy of the data. Records with obvious spatial errors were removed, and according to the latest International Union for Conservation of Nature (IUCN) standards, we identified 5 critically endangered (CR), 9 endangered (EN), 22 vulnerable (VU), 19 near threatened (NT), and 408 least concern (LC) bird species. CR, EN, VU, and NT species are considered threatened (see Data S1).

### 2.3 Analysis of data

To calculate functional rarity, we considered functional traits related to body size, reproductive traits, and feeding behavior. From the Chinese avian trait data (Wang et al., 2021), we extracted the following 11 traits: nesting site (NS), wing length (WL), tarsus length (TSL), flock status (FS), egg volume (EV), migration status (MS), primary diet (PD), body length (BYL), body mass (BM), tail length (TL), and bill length (BL). All data were tested for normality prior to statistical analyses and log10‐transformed to improve the normality of residuals when necessary. In order to test for significant differences across protection statuses, we used a paired difference *t*‐test for hypothesis testing. In order to create a phylogenetic tree for the 463 birds examined in this study, we trimmed down the overall bird phylogenetic tree obtained from BirdTree (https://birdtree.org) using the ‘Hackett All Species’ option (Jetz et al., 2012). To assess the consistency of patterns in the evolutionary history of bird communities with patterns observed in specific functional traits, we utilized the *K* statistic in the R package “phytools” to measure phylogenetic signals for different trait individual characteristics (Revell, 2012). Using the collected species trait data, we performed a Principal Coordinates Analysis (PCoA) using the “vegan” package. Based on the trait data for threatened bird species, we applied log transformation to the traits and constructed a trait network using the “igraph” package (|*r*| > .2, *p* < .05). The mean clustering coefficient and network density represented the complexity of the species trait networks, while the path length represented the associations between species (Table 1). Employed the “caper” package to calculate geographic constraints on the locations of threatened bird species. In addition, we used the “funrar” package to calculate the functional rarity of threatened bird species' traits.

**TABLE 1:** Parameter significance and assumptions for the overall topology of the trait network.

Parameters	Definition	Hypotheses for trait network features of threatened species over least concern species
Path length	The average length of the shortest path between network nodes	Lower
Clustering coefficient	Mean of clustering coefficients for all traits	Lower
Graph diameter	Diameter of a traits network is defined as the longest path of the shortest paths between any two nodes	Lower
Graph density	Graph density represents the ratio between the edges present in a graph and the maximum number of edges that the graph can contain	Higher

In order to assess the range of species, taking into account the impact of data volume on modeling performance, we used interpolation methods to create buffers of 50 km around known distribution points for species with very few records (<20 observations). We considered 19 bioclimatic variables (WorldClim, http://www.worldclim.org/) with a resolution of 30 m that are known to influence bird distribution. To address the problem of multicollinearity among these variables, we selected variables with correlation coefficients (|*r*|) <.7 and variance inflation factors (VIF) <10. Among the climatic variables were mean annual temperature, isotherm, annual range of temperature, precipitation in the wettest month, precipitation seasonality and precipitation in the coldest quarter. In order to account for anthropogenic disturbances, we calculated the Euclidean distance from observation points to primary and secondary roads, farmlands, and population density. In addition, we collected Normalized Vegetation Index (NDVI) data as a proxy for aboveground biomass and aboveground vegetation cover. To assess the impact of surface water, we measured Euclidean distances from observation sites to rivers and lakes. Refer to the Data Availability Statement for access to data. Based on three commonly used General Circulation Models (GCMs) in Asia, namely, BCC‐CSM1‐1, CCSM4, and MIROC‐ESM, we depict the future climate changes under the Representative Concentration Pathways 4.5 (RCP45) and 8.5 (RCP85) for mid‐century projections (2050). We have not considered longer time projections because uncertainty increases towards the end of the century (Baker et al., 2015). We utilize five SDM algorithms available in the Biomod2 software package (Thuiller et al., 2003) to estimate the species‐climate relationships for these threatened organisms. Projections take into account the many possibilities for future changes in land use types, and we projected the future distribution of birds after climate change under current land use conditions. The algorithms include the Generalized Linear Model, Generalized Additive Model, Boosted Regression Trees, Random Forest, and Maximum Entropy Model. The initial data is used to calibrate the model with 80% of the dataset, and the True Skill Statistic (TSS) is employed to evaluate the remaining 20% of the data. The data is randomly allocated to each subset, and this process is repeated four times to perform robust cross‐validation. To create an ensemble model using a weighted mean approach, we selected all the models with a TSS value >0.8 as the consensus method based on the weighted mean approach increases the accuracy of the model. To visually represent the distribution trends of threatened bird species, we will convert the predicted suitable habitats to a scale from 0 to 1. Combining the model distribution results, we will use the Biomod2 model to automatically generate equal intervals and divide the attribute values into sub‐ranges of equal size, resulting in four levels of distribution areas: unsuitable habitat (0, 0.2), poorly suitable habitat (0.2, 0.4), medium suitable habitat (0.4, 0.6), and highly suitable habitat (0.6, 1). Using ArcGIS software, we aim to create a suitability map for the threatened bird species.

## 3 RESULTS

### 3.1 Threatened bird species traits and functional characteristics

**FIGURE 2:**
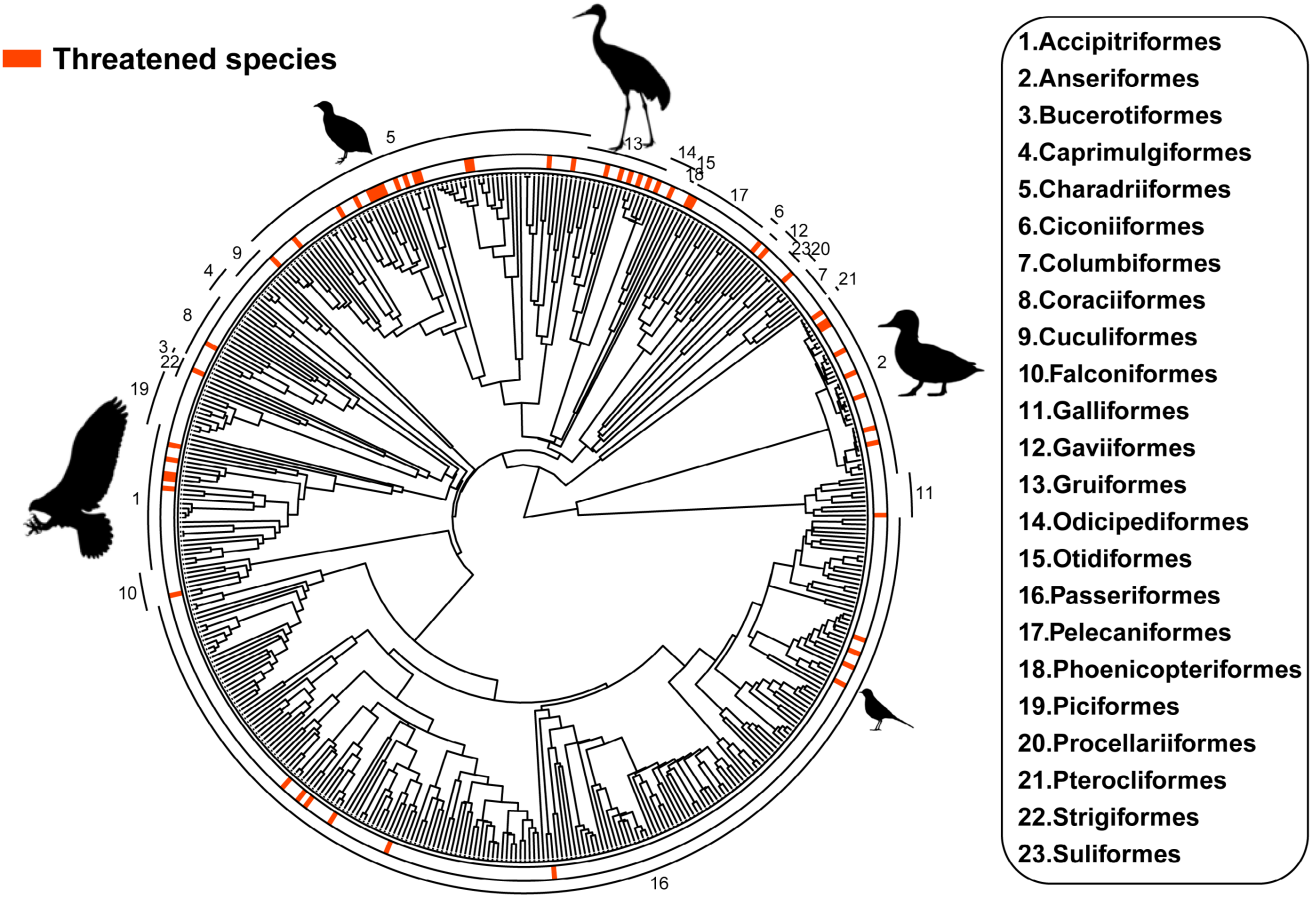
Phylogenetic evolutionary tree of threatened bird species in the Northeast region. Species receiving threats are marked in red.

Among the 63 threatened species, there is a close evolutionary relationship along the phylogenetic tree, suggesting that threatened species may have faced common environmental pressures and biological factors throughout their evolutionary history, resulting in their clustering on the evolutionary tree (Figure 2).

**FIGURE 3:**
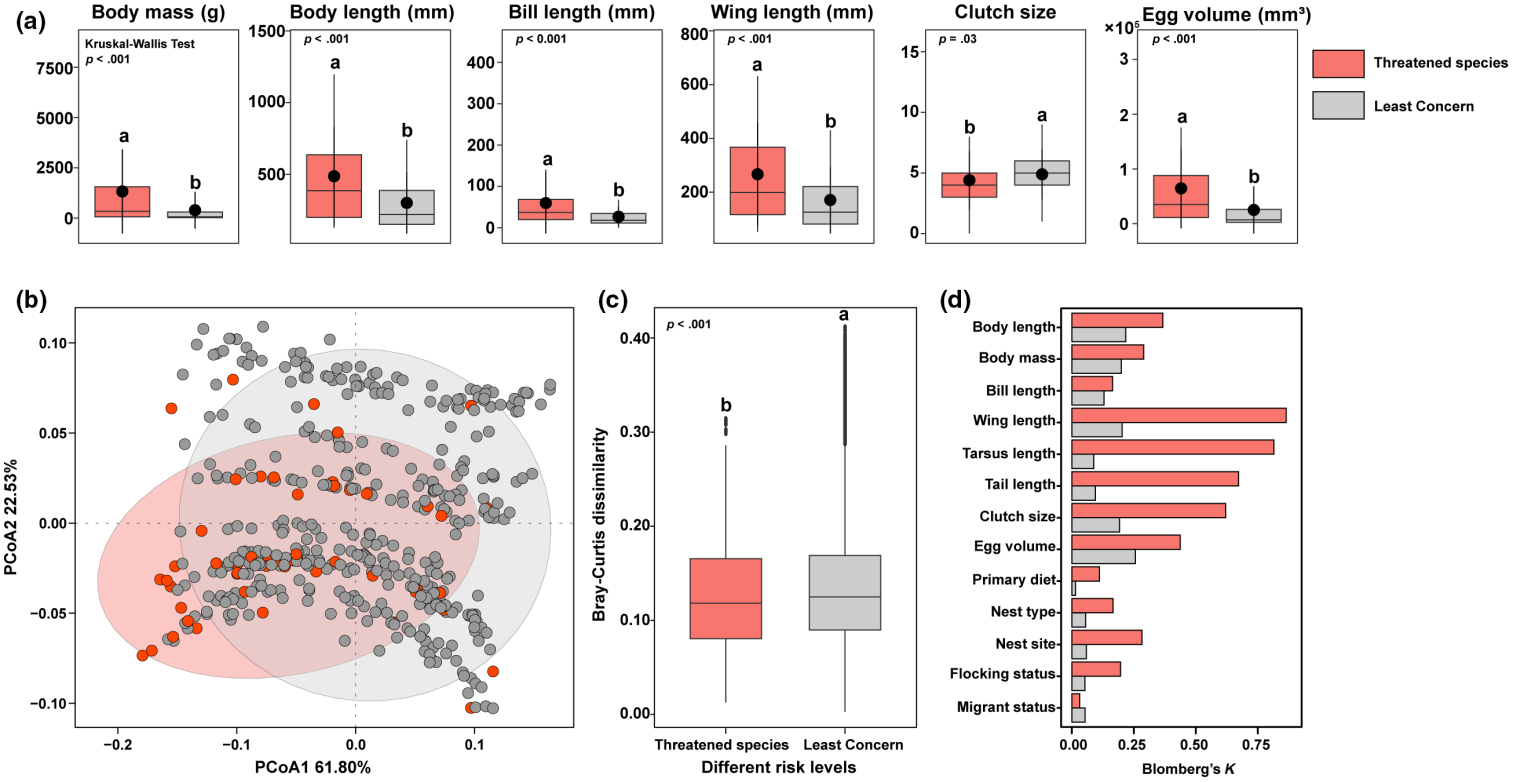
Distribution of traits among species at different threat levels, where solid black dots represent the mean values, and the boxplots show the medians. Kruskal‐test was conducted to assess the differences between groups, denoted by different letters (a). PCoA was used to represent the functional space of bird species based on their functional traits. The two axes, PCoA1 and PCoA2, explained 61.80% and 22.53% of the variance in the distance matrix, respectively (b). β‐diversity differences among different threat levels were estimated based on the Bray–Curtis distance matrix. Data denoted by different letters show significant differences between groups (*p* < .001) (c). The phylogenetic signal strength of different traits among bird species at various threat levels is depicted (d).

By analyzing the distribution of functional traits among bird species of different threat levels, we found that threatened bird species tend to have larger body sizes compared to common species. In particular, common species generally have a higher number of eggs than threatened species (Figure 3a). The PCoA results showed that two axes (PCoA1 and PCoA2) explained 61.80% and 22.53% of the variance in the distance matrix, respectively (Figure 3b). We observed that threatened species, despite being less numerous, showed a higher degree of clustering among different taxonomic groups and occupied larger ecological niches (Figure 3b). In addition, threatened species showed higher similarity in functional traits (Figure 3c, *p* < .001). We also calculated the phylogenetic signal strength for different taxonomic groups (Figure 3d). Threatened species exhibited a higher phylogenetic signal strength (Blomberg's mean *K* = 0.39) than common species (Blomberg's mean *K* = 0.12). Threatened species displayed strong phylogenetic conservatism in morphological traits, with wing length showing the highest phylogenetic signal strength (Blomberg's *K* = 0.86, common species Blomberg's *K* = 0.20). Conversely, common species showed stronger phylogenetic signal for egg volume (Blomberg's *K* = 0.26, threatened species Blomberg's *K* = 0.44).

**FIGURE 4:**
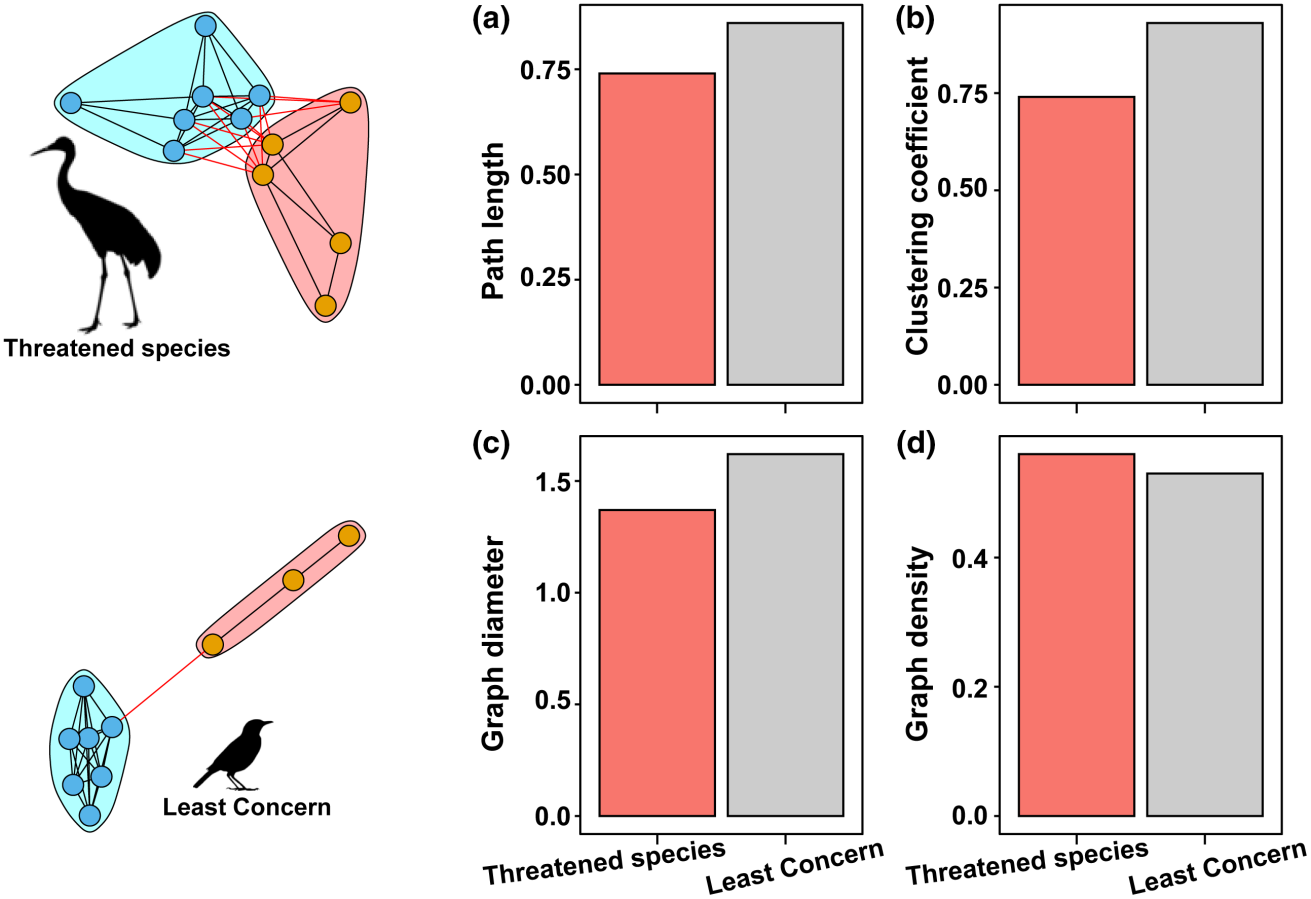
Network properties of threatened and common species' trait networks (a–d). (a) Displays the average path length within the networks, indicating a marginally higher value for Threatened species compared to species of Least Concern, suggesting a longer distance between nodes in their ecological networks. (b) Presents the clustering coefficient, showing similar levels of node clustering in networks of both Threatened species and species of Least Concern, indicating comparable local interconnectivity. In (c), the graph diameter is depicted, with Threatened species having a slightly larger diameter, pointing to more extended ranges of interaction within their networks. (d) Compares the graph density, revealing very similar connectivity levels for both groups, with no significant variation in the number of edges relative to the number of nodes in their respective networks.

Network analysis provides a new solution for understanding the complexity of the organization of species traits across ecosystems (Table 1; Figure 4). The trait correlation network of threatened bird species shows a tighter structure compared to the network of bird species of least concern. The average clustering coefficient (0.74) and network density (0.56) indicate a higher level of complexity in the threatened bird trait network (Figure 4b,d). On the other hand, the path length (0.86) is greater in the least concern species (Figure 4c), indicating lower trait correlations compared to the threatened species, indicating a less tightly connected network of trait relationships in common species.

### 3.2 Impact of climate conditions on the distribution of threatened species

**FIGURE 5:**
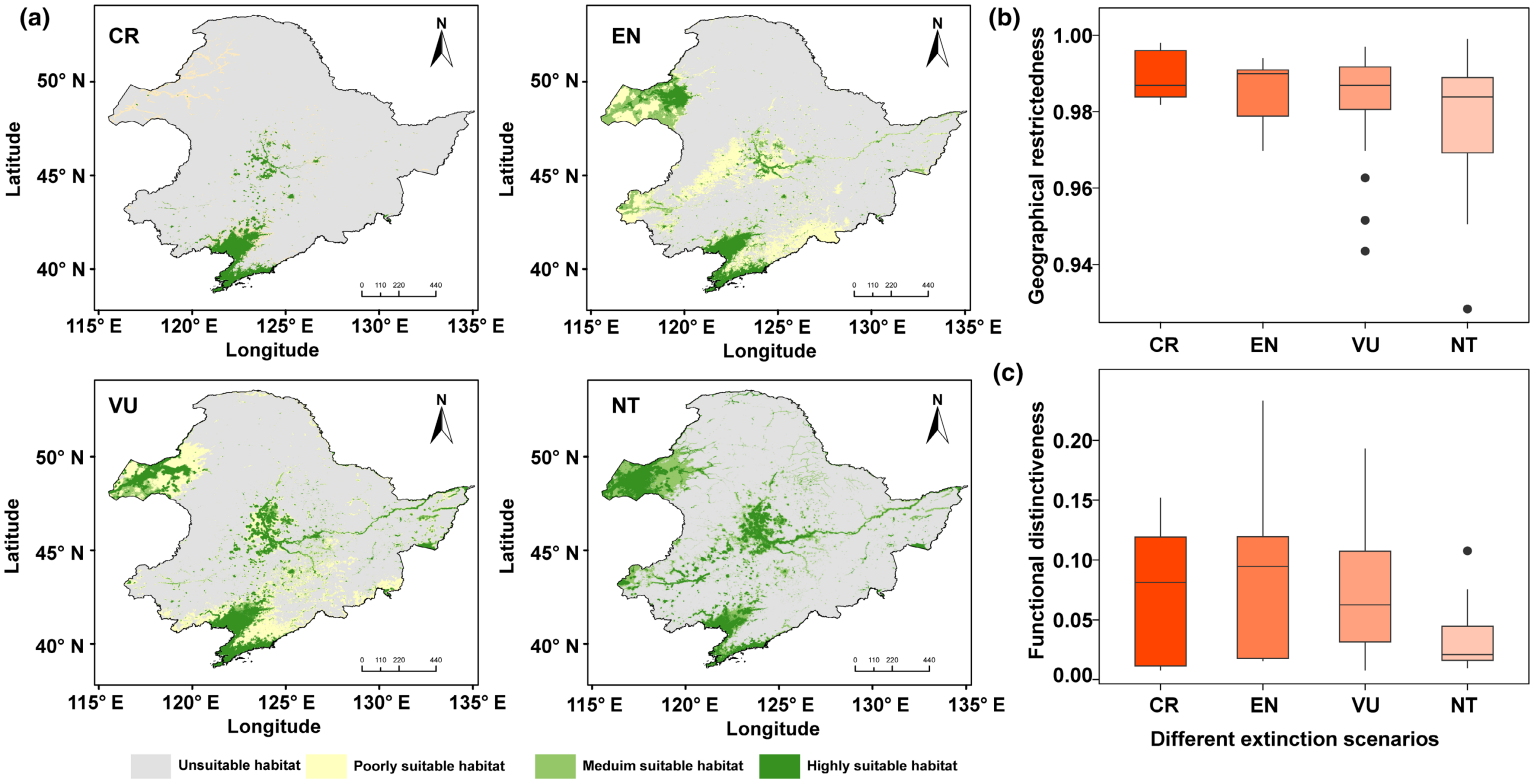
Habitat suitability and diversity metrics across different conservation status categories in the Northeast China region. (a) maps out habitat suitability across four conservation statuses—CR (Critically Endangered), EN (Endangered), VU (Vulnerable), and NT (Near Threatened)—with color gradients representing varying levels of habitat suitability from unsuitable to highly suitable. (b) and (c) provide box plots of geographical and functional distinctiveness, respectively, for each conservation status.

**FIGURE 6:**
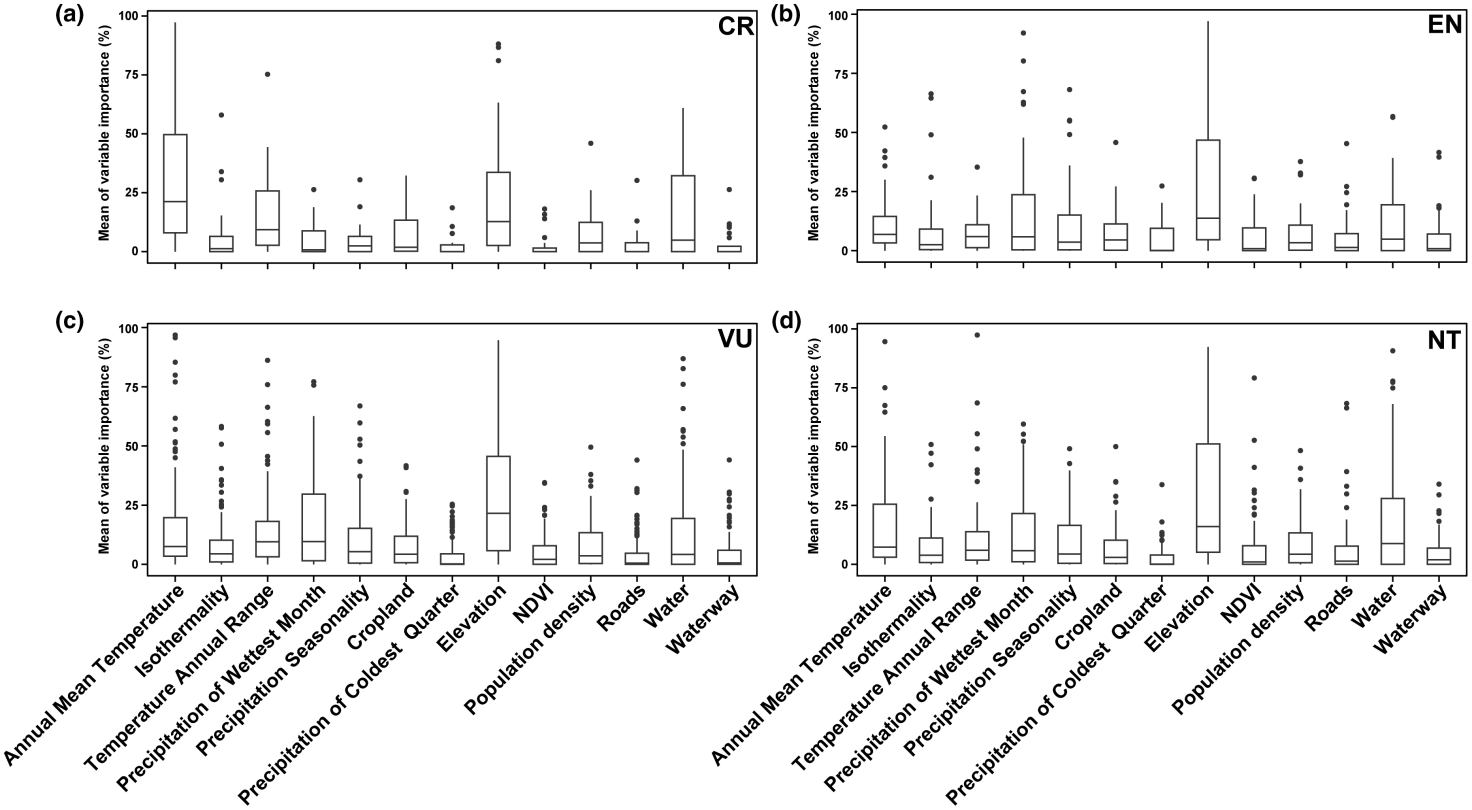
Effects of different environmental variables on threatened birds. The image displays four box plots (a–d) comparing the influence of various environmental variables on habitat suitability for species categorized by conservation status: CR (Critically Endangered), EN (Endangered), VU (Vulnerable), and NT (Near Threatened). Each plot represents a different conservation status and illustrates the range of suitability scores influenced by environmental factors such as annual mean temperature, temperature seasonality, precipitation of the wettest quarter, etc. Outliers and variability within each variable suggest differing impacts on the habitat suitability for species in each conservation category.

Different climatic conditions have a direct impact on species distribution. To identify potential geographical hotspots for threatened species, we analyzed the potential distribution areas based on current climate conditions (Figure 5). We found that threatened species show a certain spatial consistency in their distribution. In general, species diversity is higher in the southern regions and decreases towards the north. Critically endangered species are mainly distributed in coastal areas and the central region with extensive wetland distribution. For other threatened species, the western part of the Northeast region is also a potential distribution area. Among the key factors influencing the distribution of threatened species, mean annual temperature plays a critical role (30.40%, Figure 6). In addition, altitude and distribution of water are key factors influencing the distribution of endangered, vulnerable and near‐threatened bird species. Critically endangered and endangered species show higher functional uniqueness (CR: 0.086; EN: 0.074), while geographical constraints are stronger for critically endangered species (CR: 0.989) (Figure 5b,c).

**FIGURE 7:**
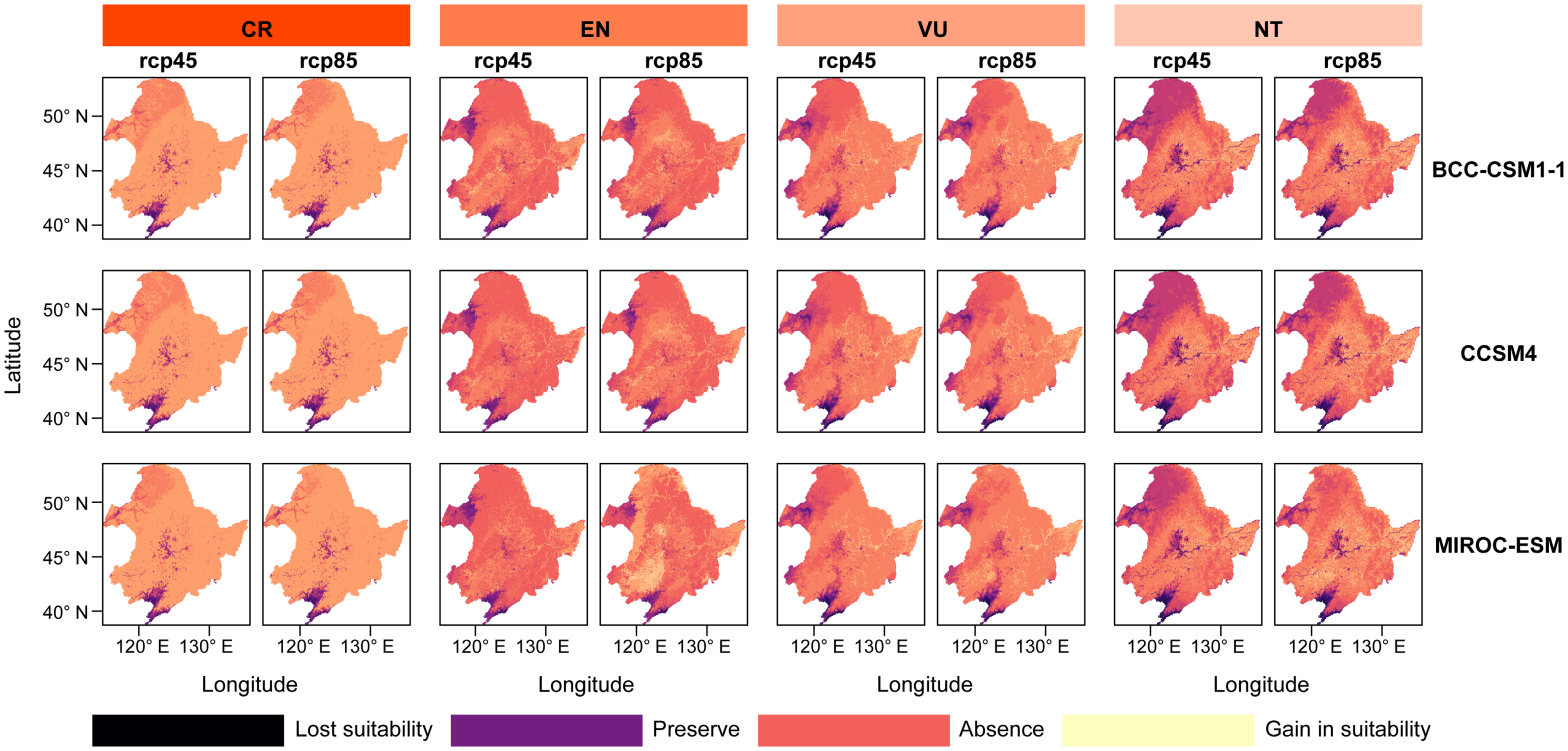
Regional changes in the distribution of threatened bird species under future climatic conditions in the Northeast China region. The series of maps showcases the projected changes in habitat suitability for species with varying conservation statuses: CR (Critically Endangered), EN (Endangered), VU (Vulnerable), and NT (Near Threatened) under two Representative Concentration Pathway (RCP) scenarios (rcp45 and rcp85) across three climate models (BCC‐CSM1‐1, CCSM4, MIROC‐ESM). Each map illustrates the spatial distribution of areas where habitat suitability is expected to be lost, preserved, absent, or gained due to climate change, with color coding indicating the nature of the change.

**FIGURE 8:**
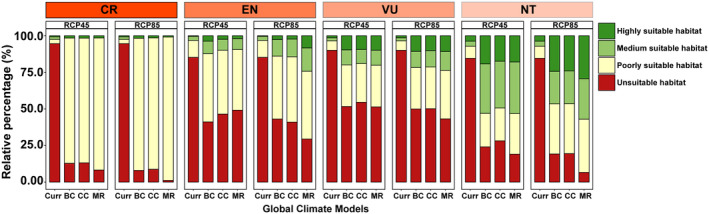
Comparative analysis of habitat suitability across conservation statuses and climate projections. This bar chart visualizes the relative percentages of habitat suitability categories for different conservation statuses: CR (Critically Endangered), EN (Endangered), VU (Vulnerable), and NT (Near Threatened) under current conditions (Curr) and two climate change scenarios (RCP4.5 and RCP8.5) predicted by three global climate models (BCC, CCSM4, MIROC‐ESM). The suitability is classified into four categories: highly suitable, medium suitable, poorly suitable, and unsuitable habitats. The colors range from green (highly suitable) to red (unsuitable), offering a stark visual representation of the potential impact of climate change on habitats for each conservation status.

Through simulations under a range of climate conditions, we found that both RCP45 and RCP85 future climate scenarios would lead to varying degrees of expansion in the suitable range of threatened bird species (Figure 7). For critically endangered bird species, the distribution of poorly suitable habitat will increase significantly under future conditions, while the distribution of highly suitable habitat will almost double (current: 0.73%; future climate: 1.49%) (Figure 8). Similarly, for EN, VU, and NT species, the future climate will significantly increase their Suitable Habitat and decrease the distribution of Unsuitable Habitat. Overall, the range of suitable habitats for bird species will be even larger under RCP85 conditions.

## 4 DISCUSSION

Over the past few decades, the Northeastern region of China has experienced significant anthropogenic disturbances, including deforestation and the expansion of agricultural activities, which have directly resulted in habitat loss for wildlife (Yang et al., 2018). Despite being warm‐blooded animals, birds in the Northeast region are still highly sensitive to temperature changes. Threatened bird species have more similar traits compared to common species, and show stronger signals of phylogenetic relatedness in certain traits. These threatened bird species also have greater functional rarity and geographic limitations. Threatened species with greater functional uniqueness may imply a greater contribution to ecosystem functioning and stability. In addition, the greater geographic restriction of threatened species indicates that their geographic range is more limited. This geographic limitation exposes threatened species to greater threats such as habitat loss, climate change, and predation.

### 4.1 Analysis of threatened bird species traits and trait rarity in Northeast China

The formation and extinction of species may lead to the diversification of traits and the blurring of functions (Urban, 2020). In the Northeastern region of China, the perennial low‐temperature environment has driven birds to evolve towards better insulation and reduced heat loss. As species form and adapt to environmental conditions, the traits of birds of different threat levels gradually converge (Figure 3b). Functional traits are the manifestations of adaptive strategies formed through the long process of evolution and interaction with the environment (McGill et al., 2006). These traits encompass various aspects, such as morphological structures, physiological characteristics, behavioral patterns, and life history strategies. Functional traits are closely related to a species' ecological functions, including survival, reproduction, and resource utilization. For instance, in plants, morphological traits like leaf area and leaf thickness can influence their photosynthetic efficiency and water‐use efficiency, thereby determining their growth and competitive abilities in different habitats (Goodness et al., 2016; Li et al., 2022). Similarly, for birds, functional traits like body size, dietary habits, and migration behavior can affect their resource acquisition, survival success, and population dispersal capabilities (Vandewalle et al., 2010). Threatened bird species exhibit larger body size and longer wing length, while common species tend to have more eggs in their nests. These differences directly reflect the functional disparities and ecological niche specialization among bird species of different threat levels and how they adapt to environmental changes and resource utilization strategies (Figure 3a). Comparing functional traits among different species can reveal key functional groups in ecosystems, the degree of ecological niche overlap among species, and the stability of ecosystem functions. Most functional traits are multifunctional and adapted to various selection pressures, making it complex to establish their integration and relative mechanism importance using traditional correlation and clustering methods (He et al., 2020). The tightness of the trait network may reflect more interactions and dependencies among threatened bird species in the ecosystem. The closer connectivity in the trait network between threatened bird species (Figure 4) might be due to shared characteristics related to interactions, habitat utilization, population features, and environmental pressures (Schmiegelow & Mönkkönen, 2002). These birds may exhibit closer species interactions, depend on each other's presence, and rely on specific ecological roles and resource utilization patterns (Wisz et al., 2013). Threatened bird populations may share similar characteristics in terms of population size, structure, and behavior, further enhancing the tightness of their trait associations. In the face of environmental pressures and stressors, threatened bird populations need to cooperate and coordinate more closely with limited resources and shared threats, possibly leading to stronger connections in the network to cope with common challenges (Schmiegelow & Mönkkönen, 2002). The threat level of species does not contradict the functional rarity of species in evolution. Although regional species are gradually converging in traits, we also found specific traits that are overexpressed in certain taxonomic levels among the currently threatened bird species in the northeastern region (Figure 3d). Previous research has suggested that the scarcity of endangered bird species may be determined by the top‐down control of food webs, such as Charadriiformes and Accipitriformes, which are highly threatened and sensitive to food resources, mainly preying on other species and occupying a large ecological niche space. Consequently, they are more susceptible to resource constraints (Loiseau et al., 2020).

### 4.2 Geographical distribution patterns and changes in suitable habitats for threatened bird species

Many threatened bird species have small geographical ranges, meaning that they are found only in specific areas or habitats (Figure 5a). This makes them particularly vulnerable to threats such as habitat loss, climate change and predation. The narrowness of their geographic range may increase their vulnerability to extinction (Gaston & Fuller, 2009). If the specific areas on which they depend are disturbed by human activities, their habitat will be severely affected. This can lead to a decline in habitat quality and a reduction in food and nesting resources, weakening the survival and reproductive capacity of threatened bird species (van de Ven et al., 2020). We found that mean annual temperature and the distribution of water systems are the most important factors influencing the distribution of threatened bird species. Birds are warm‐blooded animals and their physiological functions and metabolic activities are strongly influenced by temperature. Varying mean annual temperatures are crucial for providing suitable living and breeding conditions (Ruuskanen et al., 2021). Water provides important habitat, foraging and breeding sites for birds. Many birds rely on wetlands and aquatic vegetation in aquatic ecosystems as habitats and food resources. Regions with abundant water tend to attract more aquatic and wetland bird species (Scheiffarth et al., 2002). In addition to mean annual temperature and the distribution of water systems, altitude is also a key factor influencing the geographical distribution of birds. In general, birds adapt to different temperatures and climatic conditions at different altitudes and select suitable altitudinal ranges for survival and reproduction. Changes in elevation can affect vegetation types and plant community structures, thereby affecting the availability of food resources for birds (Rodenhouse et al., 2008; Xing et al., 2023). Geographic limitation makes threatened bird species more vulnerable to the effects of climate change. Because they are restricted to certain areas, they may not be able to migrate to other suitable locations if the climate in their area changes (Hansen et al., 2001). Due to their limited distribution range, predators have an increased likelihood of detecting and preying on these species. As the number of predators rises or refugees in their habitat decreases, the risk of mortality for these bird species also increases (Croxall et al., 2012). Therefore, gaining a deeper understanding of the ecological and evolutionary characteristics of threatened species and implementing effective measures to preserve their habitats and food resources are essential for their protection. When resources become scarce, predators become more susceptible to influence, leading to a decrease in their population size (Gardali et al., 2012; Mak et al., 2021). In general, the scarcity of species traits and geographic restrictions are the primary drivers of the formation and extinction of threatened species. Increasing evidence suggests that ongoing climate change has impacted the evolution and development of organisms (Williams et al., 2015). Taxa with limited geographic range are typically more vulnerable to changes in environmental conditions and experience greater impacts. Previous research has demonstrated that the number and diversity of threatened species are lower at high latitudes compared to tropical regions. However, due to the harsh climatic conditions at high latitudes, species in these regions face more environmental constraints (Saupe et al., 2019). Threatened bird species, characterized by their limited spatial distribution and ecological niches, are particularly vulnerable to the effects of future climate change. Under future climate scenarios, particularly the high‐emission RCP8.5 scenario, some regions that are currently unsuitable may become suitable for these species. This suggests a potential shift rather than a simple expansion of suitable habitats, indicating that species may need to adapt to or migrate towards these new suitable areas as their current habitats become less viable due to climate change. This may be attributed to birds' preference for warm and humid environments, with future climate warming and increased precipitation providing more suitable living conditions for them (Figures 7 and 8). It is important to note that when simulating and predicting future habitat suitability, threatened species may experience both winners and losers. Some species may benefit from new environmental conditions, while others may suffer losses. However, predicting the potential distribution of bird species requires considering factors such as topography, vegetation, human disturbance, and interspecific competition. Integrating biotic and abiotic factors will allow for a more effective understanding of the future potential suitable habitats for threatened bird species.

The study presents valuable insights into the traits and distributions of threatened bird species in China's Northeast region in response to climate change. However, several limitations need to be considered when interpreting the findings. Firstly, the study relies on available data, and the quality and completeness of the datasets could impact the accuracy of the analyses and predictions. Another limitation is the uncertainty associated with climate modeling, as climate models are subject to variations that could affect the accuracy of future distribution predictions. In addition, the study simplifies species interactions, overlooking complex ecological relationships that could influence the ecosystem. In future studies, which should be expanded to include multiple regions and taxa, a more comprehensive understanding of the impacts of climate change on threatened species in different ecosystems and geographic locations can be obtained.

## 5 CONCLUSION

The Northeast region offers a distinctive habitat for threatened birds owing to its persistent cold temperatures throughout the year and isolated location. Threatened species tend to be more phylogenetically related to each other on the evolutionary tree. There is a greater degree of similarity in morphological traits (e.g., wing length and egg size) among threatened species than among common species, which tend to lay more eggs. Threatened species also show greater aggregation in functional character space, occupying fewer ecological niches. Climate conditions have a direct impact on the distribution of bird species. Annual temperature has been identified as a crucial factor affecting the distribution of critically endangered species, while altitude and water distribution play critical roles for multiple threatened bird species. Based on projected future climate scenarios, it is expected that the suitable distribution areas for bird species will expand to varying degrees. In conclusion, this study sheds light on the influential factors shaping bird distribution in Northeast China and reveals characteristic traits of threatened species. These findings provide a scientific foundation for future conservation and management endeavors.

## AUTHOR CONTRIBUTIONS

**Jianwei Li:** Conceptualization (equal); formal analysis (equal); investigation (equal); methodology (equal); resources (equal); software (equal); writing – original draft (lead); writing – review and editing (lead). **Haibo Jiang:** Funding acquisition (equal); writing – review and editing (equal). **Mingjun Xie:** Data curation (equal); resources (equal). **Chuantao Song:** Data curation (equal); formal analysis (equal); resources (equal); writing – original draft (equal). **Chunguang He:** Funding acquisition (equal). **Hongfeng Bian:** Funding acquisition (lead). **Lianxi Sheng:** Conceptualization (equal); formal analysis (equal); funding acquisition (equal); project administration (equal); writing – original draft (equal).

## CONFLICT OF INTEREST STATEMENT

The authors have no relevant financial or non‐financial interests to disclose.

## Supporting information


Data S1.

## Data Availability

Relevant data and codes are provided at https://github.com/lijianweicode/Brid. All data used in this paper are freely available and downloadable from the web. Species distribution maps were provided by the Mammal Red List Assessment (http://www.iucnredlist.org/). For birds, breeding range distribution maps were extracted from China Bird Report (http://www.birdreport.cn/), Global Biodiversity Information Facility (GBIF, http://www.gbif.org/), and eBird database (eBird, https://ebird.org/). IUCN status is available on the IUCN red list (https://www.iucnredlist.org/). Population density data was obtained from https://landscan.ornl.gov/ (2022). Current and future data were obtained from WorldClim (https://worldclim.org/). The Global Roads Open Access Dataset was obtained from socioeconomic data and applications center (https://sedac.ciesin.columbia.edu/). River and water system data from OpenStreetMap (https://download.geofabrik.de/asia.html). Cropland data were obtained from the Gcographic remote sensing ecological network platform (http://www.gisrs.cn/). NDVI data were obtained from the National Science & Technology infrastructure (China 30 m annual maximum NDVI dataset 2000–2022, https://www.escience.org.cn/). The datasets generated during and/or analyzed during the current study are available from the corresponding author on reasonable request.
